# Genome Sequence of *Rhodococcus* sp. Strain RD6.2 DSM 46800, a Methanesulfonate-Degrading Strain

**DOI:** 10.1128/genomeA.00730-15

**Published:** 2015-07-16

**Authors:** Ana C. Henriques, Paolo De Marco

**Affiliations:** Instituto de Investigação e Formação Avançada em Ciências e Tecnologias da Saúde, CESPU, Paredes, Portugal

## Abstract

The complete genome sequence of a methanesulfonate-degrading strain, *Rhodococcus* sp. strain RD6.2 DSM 46800, which was isolated from a brackish marsh sediment sample, is described here. This is the first reported genome of a nonproteobacterial strain using methanesulfonate (MSA) as a sole source of carbon and energy, which does not possess the conventional MSA-monooxygenase (MSAMO).

## GENOME ANNOUNCEMENT

Methanesulfonate (MSA) is quantitatively a very relevant compound in the biogeochemical sulfur cycle (1–6). Several methylotrophic species isolated from different environments can grow using MSA (7–16). All strains analyzed so far at the molecular level contain an inducible MSA monooxygenase that oxidizes MSA to formaldehyde (7–13, 16). *Rhodococcus* sp. RD6.2 DSM 46800 is the first described non-*Proteobacterium* using MSA as a sole source of carbon and energy.

This strain was isolated on MSA from a brackish marsh sediment sample and showed resistance to several organic xenobiotics (17). Its genome was sequenced by Molecular Research LP (Shallowater, TX, USA) using the MiSeq Illumina sequencing platform. The genome coverage was 521×. Sequence reads were assembled using the NGen assembler (DNAStar, Inc.).

Thirteen contigs were generated by assembly, comprising a total of 5,573,556 bp (including 1,169 undetermined bases). One of the contigs, which was 81,713 bp long, was shown to be a circular DNA molecule with several plasmid signatures. The genome sequence was analyzed and annotated using the MicroScope platform (https://www.genoscope.cns.fr/agc/microscope/home/index.php) (18). It comprises 6,024 genomic objects (5,505 coding sequences [CDSs], 6 fragments of CDSs, 11 genes for miscellaneous RNA, 4 genes for 5S rRNA, and 2 for 16S rRNA) and 5 fragments with identity to 23S rRNA genes; 3,979 CDSs (72.28%) were categorized in at least one COG group. The G+C content is 68.4%.

The highest hits in BLASTn/BLASTp analyses (19) with genetic markers 16S rRNA, 23S rRNA, GyrB, EF-Tu, and RecA were from *Rhodococcus triatomae*, *Rhodococcus equi*, and *Rhodococcus opacus*. Average nucleotide identity (ANI) genome comparisons (http://enve-omics.ce.gatech.edu/ani/) (20) supported these associations (maximum score, 84.54% with *Rhodococcus triatomae*).

This genome contains genes involved in the tricarboxylic acid (TCA) cycle, glycolysis, gluconeogenesis, and the pentose phosphate pathway. Genes encoding components for an electron transport chain associated with aerobic respiration and oxidative phosphorylation were found, as well as those encoding dimethyl sulfoxide reductase, fumarate reductase, trimethylamine-*N*-oxide reductase, and nitrate reductase. Pathways for CO_2_ fixation are not present, while genes related to aromatic compound degradation and arsenate detoxification were found.

Regarding MSA degradation, classic methanesulfonate monooxygenase (MSAMO)-coding genes (7) were not found. However, the genome comprises a homolog of *ssuD*, a gene involved in MSA oxidation by bacteria that use it as sulfur source (21, 22); three more genes encoding putative alkanesulfonate monooxygenases, which may be involved in MSA degradation; and a *ssuCBA* operon encoding a putative alkanesulfonate uptake system (22, 23). Genes for methanol dehydrogenase were absent, while genes coding for alcohol dehydrogenase were identified, as well as those encoding *S*-(hydroxymethyl)mycothiol dehydrogenase for formaldehyde oxidation.

Genes encoding the diagnostic enzymes for serine or RuMP cycles were not found. However, we conjecture that strain RD6.2 may assimilate formaldehyde using a modified xylulose monophosphate pathway, in a fashion similar to what happens in *Mycobacterium* sp. strain JC1 (24, 25), employing a predicted transketolase (70.5% similar to dihydroxyacetone synthase from strain JC1).

The genomic features of *Rhodococcus* sp. RD62 DSM 46800 bring new insights into the utilization of MSA as a sole source of carbon and energy by a methylotrophic actinobacterium.

### Nucleotide sequence accession numbers. 

This whole-genome sequencing project has been deposited at DDBJ/EMBL/GenBank under the accession numbers CVQP01000001 to CVQP01000013.
